# National Sleep Awareness Week — March 3–10, 2013

**Published:** 2013-03-01

During March 3–10, 2013, National Sleep Awareness Week will be observed in the United States. The National Sleep Foundation recommends that adults get 7–9 hours of sleep per night (1). Receiving less sleep can pose serious consequences to health and safety. In a population-based community study, those who reported an average sleep duration of ≤6 hours (6.7%) were significantly more likely to also report that they had fallen asleep while driving than were those who reported average sleep duration of 7–9 hours (2.6%) (2).

In addition to creating a risk to public safety, self-reported insufficient sleep has been associated with adverse health behaviors, such as smoking, physical inactivity, and obesity (3). The origins of insufficient sleep can, in certain cases, begin early in life and pose lasting consequences. A retrospective cohort study found that self-reported instances of neglect or abuse during childhood (i.e., adverse childhood experiences [ACEs]) were associated with frequent insufficient sleep decades after their occurrence. Specifically, the odds of frequent insufficient sleep were 2.5 times (95% confidence interval = 2.1–3.1) greater among respondents reporting five or more ACEs than among those reporting no ACEs (4). Such findings suggest the importance of sleep as a measure of both public health and safety, as well as a marker of potential household dysfunction. Additional information regarding sleep is available at http://www.cdc.gov/sleep.
